# The effects of synbiotic supplementation on blood pressure and other maternal outcomes in pregnant mothers with mild preeclampsia: a triple-blinded randomized controlled trial

**DOI:** 10.1186/s12905-024-02922-6

**Published:** 2024-01-31

**Authors:** Rouhina Movaghar, Shamci Abbasalizadeh, Shabnam Vazifekhah, Azizeh Farshbaf-Khalili, Mahnaz Shahnazi

**Affiliations:** 1 Department of Midwifery, Faculty of Midwifery, Mahabad Branch Azad University, Mahabad, Iran; 2 Tabriz University of Medical Sciences, Women’s Health Research Center, Tabriz, Iran; 3 Uremia University of Medical Sciences, Uremia, Iran; 4 Physical Medicine and Rehabilitation Research Centre, Aging Research Institute, Tabriz University of Medical Sciences, Tabriz, IR Iran; 5 Faculty of Nursing and Midwifery, Tabriz University of Medical Sciences, Tabriz, Iran

**Keywords:** Probiotic, Synbiotic, Pre-eclampsia, Pregnancy Toxemias, Pregnancy hypertension, Pregnancy outcomes, Pregnancy complications

## Abstract

**Introduction:**

Preeclampsia affects a significant percentage of pregnancies which is a leading cause of premature birth. Probiotics have the potential to affect inflammatory factors, and oxidative stress, which are linked to the development of preeclampsia. The study aimed to compare the effect of synbiotic and placebo on blood pressure and pregnancy duration as primary outcomes, and other pregnancy outcomes.

**Methods:**

This study comprised 128 pregnant women with mild preeclampsia and gestational ages exceeding 24 weeks who were referred to the high-risk pregnancy clinic. It was a randomized, controlled, phase III, triple-blinded clinical experiment. The intervention and control groups were distributed to the participants at random. Intervention group received one oral synbiotic capsule, and control group received placebo daily until delivery. Based on gestational age at the time of diagnosis, preeclampsia was stratificated as early (< 34 weeks) or late (≥ 34 weeks). Data obtained from questionnaires, and biochemical serum factors were analyzed using SPSS software version 23 software.

**Results:**

With the exception of the history of taking vitamin D3, there were no statistically significant variations in socio-demographic variables between the research groups. After the intervention, the means of systolic blood pressure (adjusted mean difference: -13.54, 95% CI: -5.01 to -22.07), and diastolic blood pressure (adjusted mean difference: -10.30, 95% CI: -4.70 to -15.90) were significantly lower in the synbiotic-supplemented group than in the placebo group. Compared to the placebo group, the incidence of severe PE (*p* < 0.001), proteinuria (*p* = 0.044), and mean serum creatinine level (*p* = 0.005) significantly declined in the synbiotic-supplemented group after the intervention. However, our analysis found no significant association for other outcomes.

**Conclusion:**

Based on our results, synbiotic had beneficial effects on some pregnancy outcomes. Further studies with larger samples are needed to verify the advantages of synbiotic supplementation for high-risk pregnancies, particularly with regards to higher doses, and longer intervention periods.

**Trial registration:**

IRCT20110606006709N20.

## Introduction

Preeclampsia (PE) is a condition in which pregnant individuals experience high blood pressure (systolic blood pressure of 140 mmHg or higher, or diastolic blood pressure of 90 mmHg or higher *(mild PE: systolic blood pressure of 140 mmHg to Less than 160 mmHg, or diastolic blood pressure of 90 mmHg to Less than 110 mmHg)* [1], and the presence of protein in the urine. It can also lead to complications, such as kidney failure, low platelet count, impaired liver function, and fluid accumulation in the lungs [2]. This disorder affects mothers and fetuses negatively in both the short- and long-term [3] and occurs in around 3–8% of pregnancies [4]. Overall prevalence of PE in Iran is 5% [5]. Preterm birth, intrauterine growth restriction, and fetal mortality are all outcomes associated with PE. This condition is a significant factor in the need for admittance to high-risk prenatal and postnatal care facilities [6, 7].

Although it has been proposed that the administration of low doses of aspirin [8], as well as certain supplements like vitamin E and C, may serve as a preventive measure for PE in high-risk mothers, particularly those with a previous history of PE [9], there is currently no reliable and immediate method of prevention or treatment for PE. The definitive treatment at present involves parturition and the expeditious removal of placenta [10]. To prevent maternal morbidity in the case of early PE, specialists may have to terminate the pregnancy, although this might result in serious newborn morbidities. These include illnesses, such as chronic pulmonary disease, cerebral palsy [10], intracranial hemorrhage, premature retinopathy, and mortality, particularly in babies delivered before 33 weeks [11]. The precise mechanism of PE remains uncertain. It was hypothesized that a rise in adipose tissue may cause an inflammatory response, disrupting growth, and placental angiogenesis, ultimately resulting in PE [12]. Endothelial dysfunction and oxidative stress are significant contributors to the pathogenesis of preeclampsia by inducing the excessive production of pro-inflammatory mediators throughout the body [13, 14]. It can be said that PE may be associated with inflammation, which may be induced by an infection as well [15]. Gut microbiota is highly diverse, and harboring trillions of microorganisms in human digestive system. The shaping and multiplication of gut microbiome starts at birth, while the modification of their composition depends mainly on various genetic, nutritional, and environmental factors. Alterations in the composition and functionality of the gut microbiota may lead to changes in intestinal permeability, digestion, metabolism, and immunological responses. The pro-inflammatory state caused by alternation of gut microbiota balance lead to the onset of many diseases ranging from gastrointestinal and metabolic conditions to immunological, and neuropsychiatric diseases [16]. Detectable changes in the intestinal microbiota occur in PE patients from the second to third trimester of pregnancy. In PE patients, dysbiosis in the third trimester promotes inflammation, and this inflammatory axis may link the development of PE to the intestinal microbiota [17]. Unbalanced intestinal microbiota has been proposed as a potential cause of PE in the animal models of hypertension [18]. Probiotics and prebiotics are the main parts of synbiotic supplements [19]. The first group comprises viable microorganisms that may enhance well-being when eaten in appropriate quantities [20]. Prebiotics are a kind of dietary carbohydrates that cannot be digested and help to increase the growth and effectiveness of probiotics [21]. Probiotics can enhance digestive and kidney health, regulate blood pressure, prevent diabetes, and improve overall health by eradicating harmful bacteria and modulating inflammatory processes [22]. Probiotics have anti-inflammatory effects by controlling blood pressure, and inflammation-related gene expression [23], decreasing the expression of LPS on gram-negative bacteria [24, 25], and lowering inflammation in human placental trophoblast cells [25, 26]. Probiotics have potential therapeutic benefits for inflammatory conditions, including PE, based on the clinical data. Nevertheless, there is a scarcity of research on the correlation between probiotics and pregnancy outcomes in pregnant women [27]. The existing studies mostly concentrate on the preventative and protective effects of these supplements in connection to gestational hypertension and preeclampsia [28]. Based on our literature review, no clinical trial has studied probiotic or synbiotic to treat PE. This study was conducted to survey the effectiveness of oral synbiotic mi PE and preventing complications. Timely management of this condition is crucial for improved perinatal outcomes, including maternal and fetal outcomes.

## Materials and methods

### Study design and setting

It was a randomized, controlled, triple-blinded, phase III clinical trial approved under the ethics code of IR.TBZMED.REC.1398.556 by Tabriz University of Medical Sciences and registered in date of 25/09/2019 at the Iranian Registry for Clinical Trials (IRCT20110606006709N20). The protocol of this study has already been published [29].

Reports indicate that probiotic supplements are harmless and do not have any adverse effects on either the mother or the fetus [30, 31]. The research focused on pregnant women in Tabriz, Iran, who visited the high-risk pregnancy clinic at Al-Zahra Hospital. This hospital serves an area with a relatively high occurrence of PE. The women included in the study had mild PE at 24 weeks of gestation or later.

### Outcomes

Primary outcomes included systolic, and diastolic blood pressure, and the duration of pregnancy. Secondary outcomes included the incidence of severe PE, proteinuria, serum creatinine level, platelet count, and serum levels of liver enzymes (ALT, AST), bilirubin, LDH, and other outcomes that are completely stated in the protocol article of this study [29].

### Inclusion/ exclusion criteria

The inclusion criteria consisted of a single pregnancy, a gestational age of 24 weeks or more, a diagnosis of mild preeclampsia, and stable maternal and newborn circumstances that permitted a therapeutic approach including waiting, as determined by obstetricians and gynecologists. Exclusion criteria were as follows: being diagnosed with cardiovascular disease, renal, and liver failure, chronic and severe hypertension, the history of allergy to probiotics, consuming antibiotics in the past two weeks, acute gastrointestinal problems, using the glucocorticoids and immunosuppressants (except in cases where corticosteroids were prescribed to accelerate fetal lung maturation), the occurrence of maternal or fetal conditions, related or unrelated to PE, requiring immediate delivery.

### Sample size

Using G*POWER (version 3.1.2) software and considering a study power of 80%, α = 0.05, and two-tailed testing, the sample size was determined as *n* = 39 per group based on gestational age at the time of delivery, as *n* = 34 based on systolic blood pressure, as *n* = 21 based on the diastolic blood pressure [32], and as *n* = 64 based on the duration from the time of PE diagnosis to delivery [33]. Finally, regarding a 10% drop-out, the sample size was considered *n* = 128 (per group *n* = 64).

### Sample Recruitment and clinical procedures

Using the available sample technique, the study’s eligible pregnant women were enrolled. After being evaluated for eligibility, the participants received appropriate explanations of the study’s goals, procedures, risks, and benefits. After obtaining written informed consent, a basic demographic information form was completed for each participant by the researcher. One synbiotic capsule (LactoCare, cont. 10^9^ CFU, Zist Takhmir Co.) containing high amounts of probiotics (lactobacilli, bifidobacterial, and streptococci), along with fructo-oligosaccharide prebiotics (to support the growth and activity of probiotics) was daily prescribed for the participants of the intervention group.

In accordance with national policy, the treatment for mild PE includes the mother and fetus being admitted to the hospital, closely monitored, and decisions made depending on the gestational age. Primary care involves the administration of antihypertensive drugs, screening disease severity, and exacerbation (e.g., headache, visual impairment, epigastric pain, and sudden weight gain of about 1.5 kg or more per week) symptoms, measuring uterus height and gestational age, daily weighing and resting, prescribing high-protein high-calorie diet, and monitoring blood pressure every four hours. Upon admission, urine protein levels were assessed. 24-hour urine samples were taken if proteinuria (+ 1 or higher) or a protein to creatinine ratio above 0.3 were found. The renal function was evaluated by measuring the serum creatinine level in cases where proteinuria was seen in the 24-hour urine samples. There was no more testing conducted. Cell blood counting and serum level measurements were conducted multiple times per week on the mother based on the hypertension severity. Tests included platelet count, creatinine, liver enzymes (ALT, AST), bilirubin, and LDH.

Fetal health assessment involved daily monitoring of heart sounds, and fetal movements, along with regular ultrasounds to check growth, and biophysical profiles (i.e., AFI and NST). Several factors, such as the duration of pregnancy, the intensity of preeclampsia, restricted intrauterine growth, the amount of amniotic fluid, and anomalies in fetal blood vessels seen by Doppler ultrasonography, influenced the frequency of tests conducted. Regular care was given till birth if gestational age was under 37 weeks. If gestational age was 37 weeks or higher, pregnancy was terminated.

The patients with controlled blood pressure, and proteinuria were discharged, and monitored as outpatients based on specialist’s discretion. Women visited clinic once or twice a week to check their blood pressure and other parameters, and received synbiotic capsules or placebo and delivered empty envelopes. Participants continued this task until delivery. Few mothers were discharged during the study.

### Randomization and blinding

**Fig. 1:**
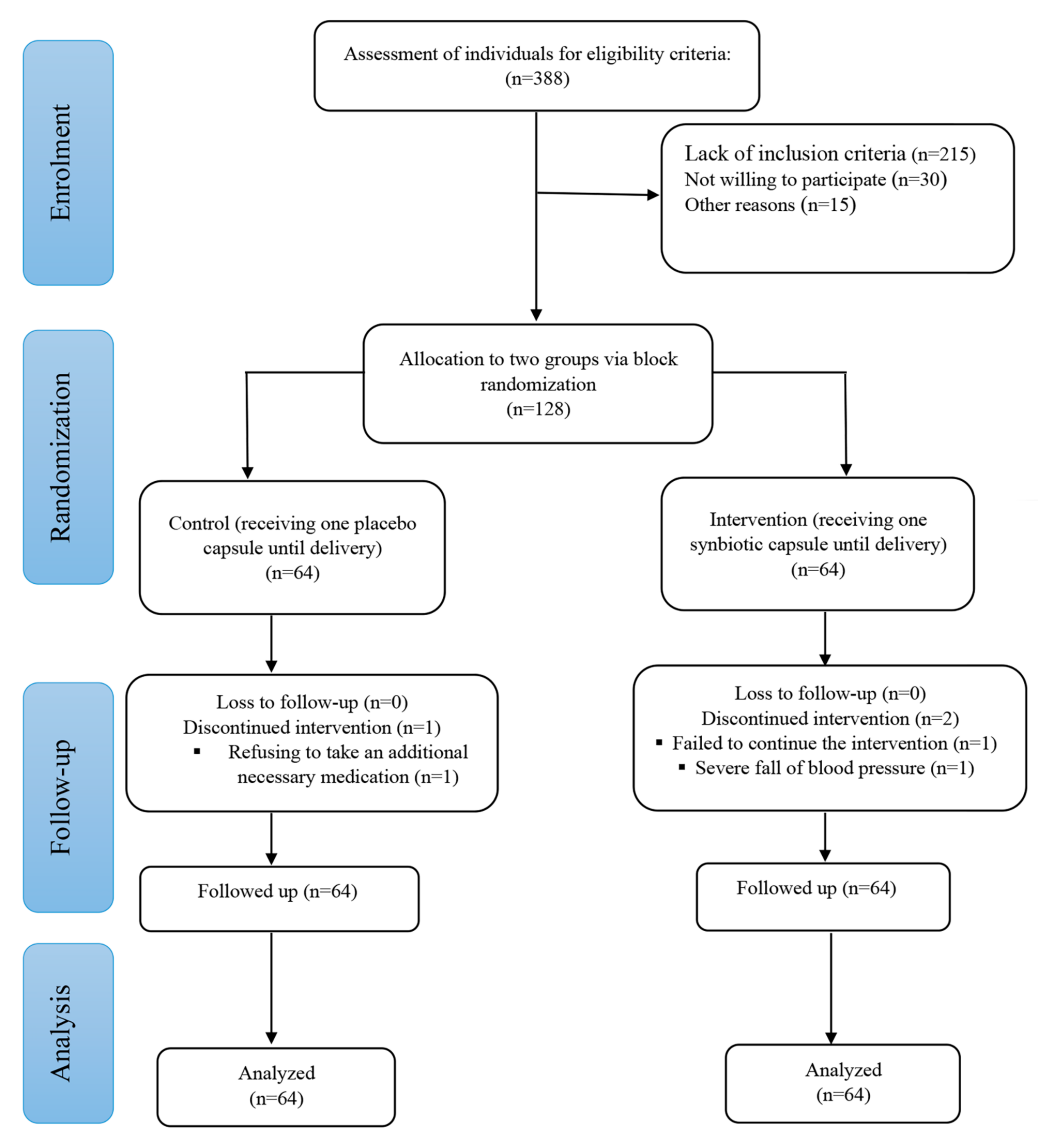
The CONSORT diagram of the study

Participants were assigned to synbiotic supplementation or placebo group using Random Allocation Software (RAS), and block randomization method. Allocation ratio was 1:1 with block sizes of four and six. Envelopes were equally prepared in the same number of participants, and numbered from 1 to 128. The envelopes were sealed and assigned a random sequence of numbers, by a not involved person. Each envelope contained either 14 synbiotic (10^9^ CFU) or placebo capsules. The participants were stratified into two groups of late and early preeclampsia in a ratio of 1:4. Envelopes were designated with numbers below 100 for early PE and numbers over 100 for late preeclampsia. The number of women in both the intervention and placebo groups was same. The first qualified individual received the first envelope, and so on until the sample size was attained. One capsule should be daily taken by participants until delivery. Comparable-looking placebo capsules were given to the control group instead of the drug. Researchers, patients, and data analysts were unaware of group assignments. Stratification was done based on gestational age at the time of PE diagnosis (i.e., early or late PE) (Fig. 1).

### Follow-up

The patients were followed up during treatment to monitor capsule consumption and side effects. Participants were told to avoid probiotic products until delivery. Throughout the research, a few patients with well-controlled blood pressure were allowed to leave the hospital. These participants received enough supply of the capsules and were instructed to check in with the clinic or a local hospital every day for blood pressure monitoring. Patients who had an increase in blood pressure were sent to the clinic. Blood pressure was also analyzed on the day of delivery. A phone number was provided to these patients after discharge so that they could contact the researcher if they had any question or problem.

### Data collection tools

These tools included a checklist for assessing eligibility criteria, a demographic information questionnaire, a checklist to document the daily consumption of medications and recording their side effects, a questionnaire for recording pregnancy, delivery, and neonatal-related information, a form for gathering the results of laboratory tests, and a data sheet for recording blood pressure. Based on the judgments of ten faculty members, the content validity method was used to approve the validity of these instruments.

### Statistical analysis

The data were inputted into SPSS software, specifically version 23. The normality of quantitative variables in each group and subgroup was assessed through using Kolmogorov-Smirnov test. To describe the data, frequency, percentage, and mean (standard deviation) were employed. To compare demographic factors between the study groups, various statistical tests were used, including Fisher’s exact test, the independent t-test, Chi-square test, trend Chi-square test, and Chi-square test. ANCOVA was implemented to compare the means of quantitative variables among the study groups, while taking into account baseline values, vitamin D consumption, BMI, and the history of pre-eclampsia as confounding variables. Logistic regression was used to compare variables with binary (categorical) outcomes among the groups, while adjusting for confounding variables. The significance level for all tests was set at 0.05, with a confidence interval of 95%. All calculations were conducted based on the intention-to-treat analysis approach. Randomization was employed to minimize the influence of confounding variables on the study outcomes.

## Results

### Participants

From February 2021 to August 2022, individuals were enrolled as research subjects (Fig. 1). Among 128 eligible patients with moderate PE, one patient from the intervention group and one from the control group discontinued the intake of the supplement. Furthermore, a member of the intervention group chose to withdraw due to experiencing hypotension. Notably, the research had no cases of attrition, and as the intention-to-treat (ITT) strategy was used throughout data analysis, all 128 mothers’ data were included at the end of the intervention period. Furthermore, the majority of participants (97.65%) successfully consumed all the provided capsules throughout the intervention period.

### Participants’ baseline characteristics

The participants in the synbiotic-supplemented group had a mean (standard deviation: SD) of age of 28.9 (4.8) years, while those in the control group had a mean age of 27.9 (4.5) years. The mean gestational age at the time of entering the study was 208.68 (27.8) days in the synbiotic group and 213.39 (22.3) days in the placebo group. The average systolic blood pressure was 134.07 (9.08) mmHg in the synbiotic group and 134.67 (8.81) mmHg in the control group. Additionally, the mean diastolic blood pressure was 83.12 (7.37) in the synbiotic group and 83.34 (5.10) in the control group. There was a significant difference between two groups in terms of the consumption of vitamin D3 during pregnancy (*p* < 0.001). No significant differences were found between the two groups in relation to other socio-demographic characteristics as shown in Table 1.

**Table 1:** The distribution of some socio-demographic features of participants

Features		Synbiotic (*n* = 64) Mean (SD)	Placebo (*n* = 64) Mean (SD)	*P*
Age (years)		28.9 (4.8)	27.9 (4.5)	0.589^†^
Pre-pregnancy weight (Kg)		75.54 (7.6)	74.64 (7.8)	0.511^†^
Height (m)		1.62 (0.0)	1.63 (0.0)	0.713^†^
Gestational age at the time of admission (days)		208.68 (27.8)	213.39 (22.3)	0.293^†^
		N (%)	N (%)	
BMI (Kg/m^2^)	< 18.5	20 (31.3)	12 (18.8)	0.159^€^
	18.5–24.9	5 (7.8)	6 (9.4)	
	25-29.9	39 (60.9)	46 (71.9)	
Educational level	Lower than diploma	32 (50.0)	36 (56.3)	0.892^‡^
	High school diploma	29 (45.3)	20 (31.3)	
	Academic	3 (4.7)	8 (12.5)	
Spouse’s educational level	Lower than diploma	34 (53.1)	33 (51.6)	0.457^‡^
	High school diploma	24 (37.5)	20 (31.3)	
	Academic	6 (9.4)	11 (17.2)	
Residency	Urban regions	43 (67.2)	45 (70.3)	0.849^€^
	Rural regions	21 (32.8)	19 (29.7)	
Occupation	Housewife	57 (89.1)	57 (89.1)	0.224^†^
	Employed	7 (10.9)	7 (10.9)	
Household income	Adequate	42 (65.6)	45 (70.3)	0.426^‡^
	Inadequate	22 (34.4)	19 (29.7)	
Positive history of preeclampsia		15 (23.4)	7 (10.9)	0.100^€^
Positive history of gestational diabetes		15 (23.4)	6 (9.4)	0.054^€^
Vit D consumption		52 (81.3)	31 (48.4)	> 0.001^€^
Number of pregnancies	Nulliparous	28 (43.8)	20 (31.3)	0.201^€^
	Multiparous	36 (56.3)	44 (68.8)	
Previous deliveries	No previous delivery	40 (62.5)	26 (40.6)	0.054^§^
	Natural delivery	16 (24.2)	25 (39.1)	
	Cesarean section	8 (13.3)	13 (20.3)	
History of abortion		16 (25.6)	14 (21.8)	0.612^€^
living child		15 (23.4)	22 (34.3)	0.809^€^
Preeclampsia	Early (< 34 weeks)	50 (49.5)	51 (50.4)	0.828^§^
	Late (≥ 34 weeks)	14 (51.8)	13 (48.1)	

*: values represent means (SD)

† independnet t-test, ‡ The Chi-square for trend, € Fisher’s exact test, §:: Chi-square

BMI: body mass index

### Primary outcomes

The average systolic blood pressure following the intervention (on the day of delivery) was recorded as 138.15 (25.85) mmHg in the synbiotic group and 153.64 (22.86) mmHg in the control group, revealing a significantly lower value in the former group (*P* = 0.002, adjusted mean difference (aMD)= -13.54, 95% CI: -5.01 to -22.07) (Table 2).

**Table 2:** The comparison of maternal outcomes between synbiotic-supplemented and placebo groups

Maternal outcomes		Synbiotic (*n* = 64) Mean (SD)	Placebo (*n* = 64) Mean (SD)	Adjusted mean difference/ (95% CI)	*p*-value^§^	*p*-value^£^
Duration of pregnancy (days)^*^		232.00 (20.78)	232.60 (21.20)	-1.99 (-9.88 to 5.89)	0.618^†^	0.87
Time from PE diagnosis to delivery (days)^*^		21.59 (24.03)	19.21 (17.29)	3.34 (-4.50 to 11.95)	0.40^†^	0.30
Systolic blood pressure^*^	pre-intervention	134.07 (9.08)	134.67 (8.81)	0.59 (-2.53 to 3.72)	0.708^±^	-
	post-intervention	138.15 (25.85)	153.64 (22.86)	-13.54 (-22.07 to -5.01)	0.002^†^	0.004
Diastolic blood pressure^*^	pre-intervention	83.12 (7.37)	83.34 (5.10)	0.21 (-2.00 to 2.44)	0.846^±^	-
	post-intervention	83.23 (18.12)	95.01 (12.45)	-10.30 (-15.90 to -4.70)	> 0.001^†^	0.001
		N (%)	N (%)	Adjusted odds ratio (95% CI)	*p*-value^*^	
Incidence of severe preeclampsia^**^		28 (43.8)	49 (76.6)	5.01 (2.04 to 12.29)	> 0.001^‡^	0.001
Premature rupture of membranes^**^		9 (14.1)	4 (6.3)	0.31 (0.79 to 1.21)	0.093^‡^	0.112
Vaginal delivery^**^		33 (36.19)	32 (25.78)	0.95 (0.37 to 2.40)	0.920^‡^	0.26
Incidence of serious complications (cerebral stroke, renal failure, HELLP syndrome, DIC, pulmonary edema)^**^		7 (11.9)	4 (6.3)	1.48 (0.30 to 7.24)	0.627^‡^	0.206
Use of antihypertensive drugs^**^		53 (82.8)	59 (92.2)	1.44 (0.43 to 4.82)	0.553^‡^	0.573

* symbol represents mean (standard deviation), ** symbol represents frequency (percentage)

^±^ Independent t-test † ANCOVA, ‡ logistic regression, ^§^adjusted for vitamin D3 consumption and BMI.

^£^ adjusted for vitamin D3, BMI, and the positive history of preeclampsia

The average (SD) of diastolic blood pressure following the intervention (on the day of delivery) was measured as 83.23 (18.12) mmHg in the synbiotic group and 95.01 (12.45) mmHg in the control group, demonstrating a significantly lower value in the former group (*P* < 0.001, aMD= -10.30, 95% CI: -4.70 to -15.90) (Table 2).

### Secondary outcomes

The occurrence rate of progressing to severe PE was notably lower (approximately five times) in the group supplemented with synbiotics compared to the placebo group (*P* < 0.001, adjusted odds ratio (OR) = 5.01, 95% CI = 2.04–12.29). Other outcomes at the conclusion of the intervention, such as early membrane rupturing, delivery mode, severe PE problems, and use of antihypertensive drugs, did not show any significant differences between the groups (Table 2). Considering the levels of PE-related indicators in the serum after the intervention, the mean (SD) of serum creatinine level (mg/dl) was 0.86 (0.01) in the placebo group and 0.79 (0.01) in the synbiotic group, which displayed a statistically significant distinction (*P* = 0.005, aMD=-0.06, 95% CI=-0.11 to -0.02). Furthermore, the intervention resulted in a significant reduction in random proteinuria (mg) in the synbiotic group compared to the placebo group (*P* = 0.004, aMD= -0.47, 95% CI= -0.92 to -0.01). After adjusting the effect of PE history, this difference was insignificant (Table 3).

**Table 3:** The comparison of some biochemical factors before and after the intervention between synbiotic and placebo groups

Biochemical factors		Synbiotic (*n* = 64) Mean (SD)	Placebo (*n* = 64) Mean (SD)	Adjusted mean difference (95% CI)	*p*-value^§^	*p*-value^£^
Platelet count (**count/ml**)	Pre-intervention	211265.62 (55177.41)	212421.87 (64776.49)	1156.25 (-19892.91 to 22205.41)	0.914^†^	-
	Post-intervention	185,785 (7004)	190,789 (6943)	-5004.56 (-25533.69 to 15524.55)	0.630^‡‘±^	0.391^£^
Creatinine (mg/dL)	Pre-intervention	0.82 (0.16)	0.80 (0.12)	-0.02 ()-0.07 to 0.02	0.350^†^	-
	Post-intervention	0.79 (0.01)	0.86 (0.01)	-0.06 (-0.11 to -0.02)	0.005^‡‘±^	0.033^£^
LDH (u/L)	Pre-intervention	385.29 (99.84)	370.98 (94.23)	-14.31 (-50.88 to 22.25)	0.439^†^	-
	Post-intervention	441.50 (17.99)	405.9 (18.17)	35.51 (-17.73 to 88.76)	0.189^‡‘±^	0.245^£^
Random proteinuria (mg)	Pre-intervention	2.07 (1.23)	2.10 (1.00)	0.30 (-0.36 to 0.42)	0.881^†^	-
	Post-intervention	1.64 (1.22)	1.92 (1.07)	-0.47 (-0.92 to 0.01)	0.044^‡‘±^	0.067^£^
ALT (u/L)	Pre-intervention	19.51 (12.67)	15.76 (11.22)	-2.47 (6.73 to 1.77)	0.252^†^	-
	Post-intervention	22.70 (22.72)	15.57 (9.80)	4.91 (-3.07 to 12.91)	0.225^‡‘±^	0.212^£^
AST (u/L)	Pre-intervention	20.34 (10.26)	19.02 (6.81)	-0.38 (-3.78 to 3.02)	0.824^†^	-
	Post-intervention	23.29 (14.24)	20.95 (16.92)	1.27 (-5/68 to 8.22)	0.717^‡‘±^	0.987^£^

All data were described as mean (standard deviation)

‡ ANCOVA, :† independent t-test, ± adjusted for vitamin D3 consumption

^§^adjusted for vitamin D3 consumption and BMI. ^£^ adjusted for vitamin D3, BMI, the positive history of preeclampsia, and baseline values

No statistically significant differences were found among the study groups in terms of other outcomes, such as the duration of pregnancy, premature rupture of membranes, mode of delivery, severe disease-related complications, use of antihypertensive medications, and blood factors such as platelet count (PLT), and serum levels of lactate dehydrogenase (LDH), alanine aminotransferase (ALT), and aspartate aminotransferase (AST), following the intervention. The supplement usage compliance rates throughout the intervention period were good, with 97% in the intervention group and 96% in the control group. None of the participants in either the intervention or placebo group reported any noticeable side effects.

## Discussion

Using synbiotic capsules (with a count of 109 CFU) was employed as a daily regimen for expectant mothers experiencing mild preeclampsia from the moment of their inclusion in the research until childbirth. Based on our comprehensive analysis of relevant literature, this particular investigation stood as the primary endeavor to explore the ramifications of synbiotic substances (comprising of the probiotic strains Lactobacillus case, L. acidophilus, L. rhamnosus, L. bulgaricus, Bifidobacterium breve, B. longum, Streptococcus thermophiles, and prebiotic along with FructoOligoSaccharides (FOS) supplements) on maternal outcomes linked to preeclampsia, as well as the associated blood indicators.

In a broad sense, the management of PE yielded advantageous outcomes. Within the confines of this particular investigation, the primary endpoints encompassed systolic and diastolic blood pressure as well as the duration of pregnancy. Remarkably, the synbiotic supplement had a significant positive effect on both systolic and diastolic blood pressure, although it did not have any impact on the duration of pregnancy. The incidence of severe PE, proteinuria, and creatinine were among the secondary outcomes that significantly improved, but other secondary outcomes like premature membrane rupture, delivery method, severe disease-related complications, antihypertensive medication use, and blood factors like platelet, lactate dehydrogenase, alanine aminotransferase, and aspartate aminotransferase were unaffected. The administration of these supplements during pregnancy was validated to be devoid of any adverse consequences in both mothers and offspring [34].

Intestinal dysbiosis may serve as a causal element in the development of hypertension. The administration of probiotics has the potential to reinstate the equilibrium of intestinal microbiota, and augment the production of metabolites implicated in the regulation of blood pressure. This consequently presents probiotics as secure and dependable therapeutic interventions to enhance the maternal outcomes in expecting women with preeclampsia [35, 36]. It has been observed that probiotic yogurt exhibits promise as a dietary supplement during pregnancy. Probiotics may assist the host by colonizing or not colonizing the digestive tract through local and/or systemic effects that can be either specific or nonspecific, direct or indirect, or both [37]. Nevertheless, the precise mechanisms by which probiotics exert their therapeutic effects remain largely ambiguous [38].

Several mechanisms have been proposed to be implicated in probiotics’ potential blood pressure-reducing effects. These mechanisms include the potential to decrease systemic inflammation [13, 14], and oxidative stress [39], as well as to stabilize the renin-angiotensin system and subsequently contribute to blood pressure regulation [40]. Furthermore, probiotics have been suggested to have a role to lower total cholesterol and low-density lipoprotein (LDL) levels [36, 41], reducing blood sugar, and modulating insulin resistance [42]. Specific concepts make reference to neuroinflammation, which has been demonstrated to play a substantial role in the development of hypertension in both human and animal models. Through the microbiota-intestine-brain axis, changes in intestinal microbiota have an impact on brain homeostasis and neuroinflammation. The decline in the relative frequency of numerous bacteria producing short-chain fatty acids (SCFAs) has been reported in animal models of hypertension. The fermentation of fibers by intestinal bacteria results in the production of SCFAs [43], which in turn can exert an influence on blood pressure by either directly promoting vasodilation or inducing the plasminogen activator inhibitor-1 (PAI-1) [44]. In the hypertensive patients, the absorption of dietary calcium suppresses both calcium-induced renin and extracellular calcium uptake, thereby reducing blood pressure [45]. Probiotics enhance the absorption of dietary calcium in the intestine via the production of SCFAs and lactic acid, which lower the pH of the intestines and increase the solubility and absorption of calcium ions [46].

New therapeutic possibilities for hypertension, in the form of probiotics and prebiotics, were understood to possess utility [47]. The ingestion of these dietary fibers has been linked to decreased cardiovascular illnesses and blood pressure in various studies [48, 49]. Gomez-Arango et al. (2016) found a negative correlation between the inflammatory marker PAI-1 [50], systolic and diastolic blood pressure, and the prevalence of butyrate-producing bacteria in the gut microbiome in a study involving overweight and obese pregnant women in their sixteenth week of pregnancy. Butyrate is manufactured from dietary fibers by bacteria within the intestinal lumen. Dietary supplements that contain probiotics and prebiotics (synbiotics) have the potential to alter the composition of the intestinal microbiome, thereby offering a new approach to helping maintain normal blood pressure and reducing inflammation during pregnancy, ultimately improving outcomes for both the mother and the newborn [51].

Numerous pieces of evidence, primarily derived from studies conducted on animal models of hypertension, have substantiated a correlation between hypertension, and the composition of the intestinal microbiota. Moreover, the administration of probiotics and prebiotics has demonstrated the ability to prevent hypertension induced by obstructive sleep apnea (OSA). However, this protective effect was not observed in mice with normal blood pressure, implying that alterations in intestinal microbial balance contribute to the development of hypertension in mouse models of OSA [43]. A comprehensive review conducted by Ejtahed et al. (2020) encompassing five meta-analysis studies involving 2703 individuals of both genders, ranging from 12 to 75 years of age, revealed that the consumption of foods and supplements containing probiotics (administered for a duration of 3 to 24 weeks, encompassing multiple species, and at doses exceeding 1011 colony-forming units) effectively regulated blood pressure in adults diagnosed with hypertension (blood pressure ≥ 130/85 mmHg). These favorable effects on blood pressure may be attributed to the cumulative or cooperative impact of various probiotic species administered at high doses [52]. In this research, we did not evaluate dose-dependent effects. Previous investigations in intervention with varied dosages, however, found that high doses had a more significant impact [53]. Moreover, Tanida and colleagues showed that the prolonged intake of probiotics, namely L. Gasseri in conjunction with L. Fermentum or L. Coryniformis, exhibited a significant decline in endothelial dysfunction, oxidative stress, and vascular inflammation in murine subjects [54].

In a recent investigation, Hajifaraji et al. (2017) conducted an evaluation on the effects of probiotic capsules, which contained L. acidophilus LA-5, Bifidobacterium BB-12, S. thermophilus STY-31, and L. delbrueckii bulgaricus LBY-27, administered at a dosage exceeding 4 × 109 Colony-Forming Units (CFU), on the levels of both systolic and diastolic blood pressure in pregnant women affected by gestational diabetes mellitus (GDM). During pregnancy, the probiotic supplement prevented an increase in blood pressure; however, this effect did not become evident until six to eight weeks of supplementation. This suggests that probiotics might possess enduring beneficial properties. Furthermore, the ingestion of this supplement for a duration of eight weeks resulted in a decrease in systolic blood pressure of up to 8.7 mmHg, and diastolic blood pressure of up to 10.61 mm Hg [27]. Similarly, in a separate investigation conducted by Nabhani et al. (2018), using a synbiotic supplement exhibited the capability to lower systolic blood pressure by 9.7 mmHg and diastolic blood pressure by 4.8 mmHg [55]. The discrepancies in blood pressure levels observed across various studies may be attributed to variations in supplement dosages, durations of consumption, and characteristics of the studied populations. Although the findings of these studies align with our own research, none of them were specifically conducted as a treatment for PE.

In contrast to previous investigations, a comprehensive analysis revealed that the introduction of probiotic supplementation did not yield any discernible impact on pregnancy outcomes, particularly in relation to the blood pressure of expectant mothers afflicted with gestational diabetes [56]. Correspondingly, the outcomes of an alternative study failed to indicate any significant association between the administration of probiotics, and pregnancy outcomes, including blood pressure levels, in women diagnosed with GDM [57]. The contradictory findings might be caused by a number of variables. The fact that the underlying ailment in our individuals was different from the disorders evaluated in related publications was a significant distinction between our investigation and other studies. In contrast to the expectant women with GDM who participated in the aforementioned studies, our subjects had mild PE. An additional variable that may account for the observed discrepancies among these results is the use of different probiotic strains and varying durations of ingestion. It appears that the incorporation of probiotic-containing supplements and nourishments can yield more favorable health outcomes when employed over an extended period, thereby engendering gradual restorative effects on the intestinal microbiota.

Evidence indicates that excessive inflammatory responses may have a significant impact on the development of PE [58]. Thus, endothelial dysfunction induced by oxidative stress and systemic inflammation are crucial determinants of PE [13, 14]. Furthermore, an increase in oxidative stress during pregnancy was associated with various unfavorable outcomes, including PE [39], low birth weight [59], preterm delivery [60], and thrombocytopenia [61]. Probiotics were shown to enhance PE by mitigating systemic inflammation [62, 63] and oxidative stress [64]. A meta-analysis revealed that probiotics may not be as effective as synbiotics in lowering inflammatory variables [65]. According to a meta-analysis of six studies with 426 individuals, probiotic and/or synbiotic treatment reduced blood creatinine levels. It is suggested probiotic may improve renal function via increasing anaerobic bacteria, such as Lactobacillus and Bifidobacterium leading to decrease in PH and urea levels [66]. These probiotics effectively improved kidney parameters, such as creatinine, proteinuria, and blood pressure in mothers with mild PE. Given the significance of these factors in the diagnosis and management of PE, the occurrence of severe PE was prevented through this mechanism. However, our analysis found no significant association for liver biomarkers.

The study conducted by Nordqvist et al. (2018) analyzed the timing of probiotic milk intake during pregnancy and its potential effect on the occurrence of PE and preterm delivery. Consuming probiotic milk in the latter stages of pregnancy considerably lowered the chance of PE [28], according to the data. This protective association between the consumption of milk products containing probiotics and PE, particularly severe PE, was further demonstrated in a separate cohort study conducted by Brantsæter et al. (2011). Thus, this association was attributed to the inflammatory changes induced by probiotics, and its effect was more noticeable in cases of severe PE. Furthermore, the results indicated a dose-dependent protection against PE [67].

Although the exact biological mechanism behind the correlation of these dietary components with the PE is still not fully understood, it is possible that it is linked to the alteration of immune responses, oxidative stress, and inflammatory processes that occur during pregnancy [68].

In a systematic review conducted by Lindsay et al. (2013), the findings demonstrated a significant reduction in fasting glucose levels, the occurrence of gestational diabetes mellitus (GDM), PE, and C-reactive protein levels with the use of probiotics during pregnancy [24]. Despite these findings, there was no statistically significant difference in the occurrence of PE between the groups studied in the clinical trial by Lindsay et al. (2014) that looked at the effects of probiotics on overweight mothers [69]. The changes in gut microbiota composition among pregnant women based on their weight status were reported [70, 71]. These conflicting differences are likely attributed to confounding factors, such as obesity, which can influence gut microbiota [72] and potentially explain the disparities in outcomes observed across studies.

We can assert that the primary distinction between the prior studies and our investigation is that our investigation was executed as a meticulously regulated clinical trial for the management of PE in humans, and consequently, its effect on the outcomes connected to PE was examined. However, in earlier studies, the consequences of pregnancy were examined subsequent to the preventative intervention or in mothers with GDM or obesity or on animals. The advantages of using probiotics extend to the enhancement of metabolism, reduction of inflammation, prevention of infection, and the amelioration of pregnancy outcomes via the reduction of preterm births [38]. There were no studies that showed probiotic use to substantially lengthen pregnancy, which was the study’s secondary main outcome. These findings have also been supported by subsequent systematic review studies [29, 73]. Pregnancy length may be shortened by a number of PE and hypertension-related conditions. It is possible that the limited duration of supplement intake could not produce a significant positive impact on pregnancy length. As previously stated, the absence of notable impact on alternative outcomes in the present study, and the presence of discrepancies between the findings of this investigation and other investigations may be attributed to the characteristics of the participants.

Using the most optimal randomized clinical trial methodology, the obligatory study-specific training, the emphasis on voluntary participation, the provision of ample opportunity for decision-making, and consultation with a personal gynecologist and spouse are among the notable attributes of the study. To mitigate the loss of participants, contact via telephone was also established, and the capsules possess a simplified usability. The sample recruitment center, Al-Zahra Hospital in Tabriz, is a well-known academic institution that receives a significant number of referrals for high-risk pregnancies from a range of geographic locations and social groups that include different age ranges and socioeconomic classes. Within this establishment, the management of PE is governed by national protocols. All these factors serve to enhance the applicability of our findings.

One of the limitations encountered in the current study was the inherent nature of disease, as well as its management, which consequently restricted the opportunity for sufficient consumption of supplements among certain participants. We conducted blood pressure measurements throughout the course of the treatment. Owing to the variability of the participants’ illnesses and the varying duration of therapy, several individuals had intervention for a few days while others received treatment for a longer period. Therefore, the obtained blood pressure via the antenatal course data were unanalyzable. This is one of the limitations of the study. Due to the fact that the supplement was ingested from the moment of PE diagnosis up until delivery, and considering that PE (particularly late PE) is a tumultuous condition that can rapidly escalate and necessitate delivery, certain participants (e.g., mothers with late-onset PE) were unable to adequately partake in supplement intake. Conversely, the effects of probiotics gradually attain an optimal state [27], which may explain the lack of significant effects observed in certain pregnancy outcomes. Preeclampsia is more common in women with high BMIs [74], and there is a clear correlation between high BMI and cesarean birth [75]. It is suggested that the relationship between BMI of women who have PE and the rate of cesarean section should be carefully investigated.

## Conclusion

Drawing upon the findings of this study, it can be deduced that the administration of synbiotic supplements shows the potential to ameliorate indicators associated with PE, namely systolic and diastolic blood pressure, proteinuria, and serum creatinine levels, thereby averting the progression towards severe PE. As a result, by improving the outcomes for both mothers and newborns, this strategy shows promise as a treatment option for PE management. Even with their significant effects, these supplements have little effect on the length of gestation. To affirm the protective effects of synbiotic against the deleterious pregnancy consequences of PE, further studies of greater sample size and appropriate supplementation duration are warranted. Future studies should prioritize the evaluation of this intervention on expectant mothers afflicted with early mild PE.

## Data Availability

The datasets used and/or analyzed in the current study are available from the corresponding author on reasonable request.

## Declarations

### Access to data and results

The research group will convey the results to participants, healthcare and public health professional, and other relevant groups through publication.

## References

[1] Bennett P, Williamson C, Sykes L, MacIntyre DA, Dixon PH. Basic Science in Obstetrics and Gynaecology E-Book: A Textbook for MRCOG Part 1. Elsevier Health Sciences; 2022.

[2] Harlow FH, Brown MA (2001). The diversity of diagnoses of preeclampsia. Hypertens Pregnancy.

[3] Leeman L, Fontaine P (2008). Hypertensive disorders of pregnancy. Am Family Phys.

[4] Duley L, editor. Editor the global impact of pre-eclampsia and eclampsia. Seminars in perinatology. Elsevier; 2009. 10.1053/j.semperi.2009.02.010 19464502

[5] Kharaghani R, Cheraghi Z, Esfahani BO, Mohammadian Z, Nooreldinc RS (2016). Prevalence of preeclampsia and eclampsia in Iran. Arch Iran Med.

[6] Bilano VL, Ota E, Ganchimeg T, Mori R, Souza JP (2014). Risk factors of pre-eclampsia/eclampsia and its adverse outcomes in low-and middle-income countries: a WHO secondary analysis. PLoS ONE.

[7] Kuchake VG, Kolhe SG, Dighore PN, Patil S (2010). Maternal and neonatal outcomes in preeclampsia syndrome. Int J Pharm Sci Res.

[8] Atallah A, Lecarpentier E, Goffinet F, Doret-Dion M, Gaucherand P, Tsatsaris V (2017). Aspirin for prevention of preeclampsia. Drugs.

[9] Cardoso PM, Surve S (2016). The effect of vitamin E and vitamin C on the prevention of preeclampsia and newborn outcome: a case–control study. J Obstet Gynecol India.

[10] Bezerra Maia e Holanda, Moura S, Marques Lopes L, Murthi P, da Silva Costa F. Prevention of preeclampsia. Journal of pregnancy. 2012;2012. 10.1155/2012/435090 PMC3534321 23316362

[11] Cluver CA, Walker SP, Mol BW, Theron GB, Hall DR, Hiscock R (2015). Double blind, randomised, placebo-controlled trial to evaluate the efficacy of esomeprazole to treat early onset pre-eclampsia (PIE trial): a study protocol. BMJ open.

[12] Olson KN, Redman LM, Sones JL (2019). Obesity complements preeclampsia. Physiol Genom.

[13] Morken N-H, Vogel I, Kallen K, Skjærven R, Langhoff-Roos J, Kesmodel US (2008). Reference population for international comparisons and time trend surveillance of preterm delivery proportions in three countries. BMC Womens Health.

[14] Goswami D, Tannetta D, Magee L, Fuchisawa A, Redman C, Sargent I (2006). Excess syncytiotrophoblast microparticle shedding is a feature of early-onset pre-eclampsia, but not normotensive intrauterine growth restriction. Placenta.

[15] Yan L, Jin Y, Hang H, Yan B. The association between urinary tract infection during pregnancy and preeclampsia: a meta-analysis. Medicine. 2018;97(36). 10.1097/MD.0000000000012192 PMC6133609 30200124

[16] Gomaa EZ (2020). Human gut microbiota/microbiome in health and diseases: a review. Antonie Van Leeuwenhoek.

[17] Wang J, Shi Z-H, Yang J, Wei Y, Wang X-Y, Zhao Y-Y (2020). Gut microbiota dysbiosis in preeclampsia patients in the second and third trimesters. Chin Med J.

[18] Adnan S, Nelson JW, Ajami NJ, Venna VR, Petrosino JF, Bryan RM (2017). Alterations in the gut microbiota can elicit hypertension in rats. Physiol Genom.

[19] De Vrese M. Schrezenmeir. Probiotics, prebiotics, and synbiotics. Food Biotechnol. 2008:1–66. 10.1007/10_2008_097 18461293

[20] Behnsen J, Deriu E, Sassone-Corsi M, Raffatellu M (2013). Probiotics: properties, examples, and specific applications. Cold Spring Harbor Perspectives in Medicine.

[21] Fuentes-Zaragoza E, Sánchez‐Zapata E, Sendra E, Sayas E, Navarro C, Fernández‐López J (2011). Resistant starch as prebiotic: a review. Starch‐Stärke.

[22] Lye H-S, Kuan C-Y, Ewe J-A, Fung W-Y, Liong M-T (2009). The improvement of hypertension by probiotics: effects on cholesterol, diabetes, renin, and phytoestrogens. Int J Mol Sci.

[23] van Baarlen P, Troost F, van der Meer C, Hooiveld G, Boekschoten M, Brummer RJ (2011). Human mucosal in vivo transcriptome responses to three lactobacilli indicate how probiotics may modulate human cellular pathways. Proc Natl Acad Sci.

[24] Lindsay KL, Walsh CA, Brennan L, McAuliffe FM (2013). Probiotics in pregnancy and maternal outcomes: a systematic review. J Maternal-Fetal Neonatal Med.

[25] Yeganegi M, Watson CS, Martins A, Kim SO, Reid G, Challis JR (2009). Effect of Lactobacillus rhamnosus GR-1 supernatant and fetal sex on lipopolysaccharide-induced cytokine and prostaglandin-regulating enzymes in human placental trophoblast cells: implications for treatment of bacterial vaginosis and prevention of preterm labor. Am J Obstet Gynecol.

[26] Bloise E, Torricelli M, Novembri R, Borges L, Carrarelli P, Reis F (2010). Heat-killed Lactobacillus rhamnosus GG modulates urocortin and cytokine release in primary trophoblast cells. Placenta.

[27] Hajifaraji M, Jahanjou F, Abbasalizadeh F, Aghamohammadzadeh N, Abbasi MM, Dolatkhah N. Effect of Probiotic supplementation on blood pressure of females with gestational diabetes Mellitus: a Randomized double blind controlled clinical trial. Iran Red Crescent Med J. 2017;19(6).

[28] Nordqvist M, Jacobsson B, Brantsæter A-L, Myhre R, Nilsson S, Sengpiel V (2018). Timing of probiotic milk consumption during pregnancy and effects on the incidence of preeclampsia and preterm delivery: a prospective observational cohort study in Norway. BMJ open.

[29] Movaghar R, Abbasalizadeh S, Farshbaf-Khalili A, Shahnazi M. Effect of synbiotic supplementation on maternal and neonataloutcomes in pregnant women with pre-eclampsia: study protocol for a triple blind randomized controlled clinical trial.Crescent J Med Biol Sci. 2023;11(1).

[30] Elias J, Bozzo P, Einarson A (2011). Are probiotics safe for use during pregnancy and lactation?. Can Fam Physician.

[31] Lee J, Han J, Choi J, Ahn H, Lee S, Kim M (2012). Pregnancy outcome after exposure to the probiotic Lactobacillus in early pregnancy. J Obstet Gynaecol.

[32] Chappell LC, Seed PT, Briley AL, Kelly FJ, Lee R, Hunt BJ (1999). Effect of antioxidants on the occurrence of pre-eclampsia in women at increased risk: a randomised trial. The Lancet.

[33] Cluver CA, Hannan NJ, van Papendorp E, Hiscock R, Beard S, Mol BW (2018). Esomeprazole to treat women with preterm preeclampsia: a randomized placebo controlled trial. Am J Obstet Gynecol.

[34] Luoto R, Laitinen K, Nermes M, Isolauri E (2010). Impact of maternal probiotic-supplemented dietary counselling on pregnancy outcome and prenatal and postnatal growth: a double-blind, placebo-controlled study. Br J Nutr.

[35] Broomfield H, Harris M, Goldie J (2022). Could probiotic supplements be an effective intervention to reduce hypertension? A systematic literature review. Online J Complement Altern Med.

[36] He J, Zhang F, Han Y. Effect of probiotics on lipid profiles and blood pressure in patients with type 2 diabetes: a meta-analysis of RCTs. Medicine. 2017;96(51). 10.1097/MD.0000000000009166 PMC5758152 29390450

[37] Shenderov B (2011). Probiotic (symbiotic) bacterial languages. Anaerobe.

[38] He A, Chin J, Lomiguen CM. Benefits of probiotic yogurt consumption on maternal health and pregnancy outcomes: a systematic review. Cureus. 2020;12(7). 10.7759/cureus.9408 PMC7449615 32864237

[39] Walsh SW (2009). Plasma from preeclamptic women stimulates transendothelial migration of neutrophils. Reproductive Sci.

[40] Ong L, Shah NP (2008). Release and identification of angiotensin-converting enzyme-inhibitory peptides as influenced by ripening temperatures and probiotic adjuncts in Cheddar cheeses. LWT-Food Sci Technol.

[41] Guo Z, Liu X, Zhang Q, Shen Z, Tian F, Zhang H (2011). Influence of consumption of probiotics on the plasma lipid profile: a meta-analysis of randomised controlled trials. Nutr Metabolism Cardiovasc Dis.

[42] Tabuchi M, Ozaki M, Tamura A, Yamada N, Ishida T, Hosoda M (2003). Antidiabetic effect of Lactobacillus GG in streptozotocin-induced diabetic rats. Biosci Biotechnol Biochem.

[43] Ganesh BP, Nelson JW, Eskew JR, Ganesan A, Ajami NJ, Petrosino JF (2018). Prebiotics, probiotics, and acetate supplementation prevent hypertension in a model of obstructive sleep apnea. Hypertension.

[44] Mortensen F, Jørgensen B, Christiansen H, Sloth-Nielsen J, Wolff B, Hessov I (2000). Short-chain fatty acid enemas stimulate plasminogen activator inhibitor-1 after abdominal aortic graft surgery: a double-blinded, placebo-controlled study. Thromb Res.

[45] Resnick LM (1999). The role of dietary calcium in hypertension: a hierarchal overview. Am J Hypertens.

[46] Parvaneh K, Jamaluddin R, Karimi G, Erfani R. Effect of probiotics supplementation on bone mineral content and bone mass density. Sci World J. 2014;2014. 10.1155/2014/595962 PMC3920759 24587733

[47] Muralitharan RR, Jama HA, Xie L, Peh A, Snelson M, Marques FZ (2020). Microbial peer pressure: the role of the gut microbiota in hypertension and its complications. Hypertension.

[48] Whelton SP, Hyre AD, Pedersen B, Yi Y, Whelton PK, He J. Effect of dietary fiber intake on blood pressure: a meta-analysis of randomized, controlled clinical trials. LWW; 2005. pp. 475–81. 10.1097/01.hjh.0000160199.51158.cf 15716684

[49] Wang X, Ouyang Y, Liu J, Zhu M, Zhao G, Bao W et al. Fruit and vegetable consumption and mortality from all causes, cardiovascular disease, and cancer: systematic review and dose-response meta-analysis of prospective cohort studies. BMJ. 2014;349. 10.1136/bmj.g4490 PMC4115152 25073782

[50] Gomez-Arango LF, Barrett HL, McIntyre HD, Callaway LK, Morrison M, Dekker Nitert M (2016). Increased systolic and diastolic blood pressure is associated with altered gut microbiota composition and butyrate production in early pregnancy. Hypertension.

[51] Vital M, Howe AC, Tiedje JM (2014). Revealing the bacterial butyrate synthesis pathways by analyzing (meta) genomic data. MBio.

[52] Ejtahed H-S, Ardeshirlarijani E, Tabatabaei-Malazy O, Hoseini-Tavassol Z, Hasani-Ranjbar S, Soroush A-R (2020). Effect of probiotic foods and supplements on blood pressure: a systematic review of meta-analyses studies of controlled trials. J Diabetes Metabolic Disorders.

[53] Szulińska M, Łoniewski I, Van Hemert S, Sobieska M, Bogdański P (2018). Dose-dependent effects of multispecies probiotic supplementation on the lipopolysaccharide (LPS) level and cardiometabolic profile in obese postmenopausal women: a 12-week randomized clinical trial. Nutrients.

[54] Tanida M, Yamano T, Maeda K, Okumura N, Fukushima Y, Nagai K (2005). Effects of intraduodenal injection of Lactobacillus johnsonii La1 on renal sympathetic nerve activity and blood pressure in urethane-anesthetized rats. Neurosci Lett.

[55] Nabhani Z, Hezaveh SJG, Razmpoosh E, Asghari-Jafarabadi M, Gargari BP (2018). The effects of synbiotic supplementation on insulin resistance/sensitivity, lipid profile and total antioxidant capacity in women with gestational diabetes mellitus: a randomized double blind placebo controlled clinical trial. Diabetes Res Clin Pract.

[56] Movaghar R, Farshbaf-Khalili A, MirzaRezaei ME, Shahnazi M (2022). The Effect of Probiotics or Synbiotics on the Hypertensive disorders of pregnant women with gestational diabetes: a systematic review and Meta-analysis. J Caring Sci.

[57] Callaway LK, McIntyre HD, Barrett HL, Foxcroft K, Tremellen A, Lingwood BE (2019). Probiotics for the prevention of gestational diabetes mellitus in overweight and obese women: findings from the SPRING double-blind randomized controlled trial. Diabetes Care.

[58] Dusse LM, Rios DR, Pinheiro MB, Cooper AJ, Lwaleed BA (2011). Pre-eclampsia: relationship between coagulation, fibrinolysis and inflammation. Clin Chim Acta.

[59] Gupta P, Narang M, Banerjee B, Basu S (2004). Oxidative stress in term small for gestational age neonates born to undernourished mothers: a case control study. BMC Pediatr.

[60] Baydas G, Karatas F, Gursu MF, Bozkurt HA, Ilhan N, Yasar A (2002). Antioxidant vitamin levels in term and preterm infants and their relation to maternal vitamin status. Arch Med Res.

[61] El-Sayed AA, Preeclampsia (2017). A review of the pathogenesis and possible management strategies based on its pathophysiological derangements. Taiwan J Obstet Gynecol.

[62] Richards JL, Yap YA, McLeod KH, Mackay CR, Mariño E (2016). Dietary metabolites and the gut microbiota: an alternative approach to control inflammatory and autoimmune diseases. Clin Translational Immunol.

[63] Patel B, Kumar P, Banerjee R, Basu M, Pal A, Samanta M (2016). Lactobacillus acidophilus attenuates Aeromonas hydrophila induced cytotoxicity in catla thymus macrophages by modulating oxidative stress and inflammation. Mol Immunol.

[64] Kullisaar T, Songisepp E, Mikelsaar M, Zilmer K, Vihalemm T, Zilmer M (2003). Antioxidative probiotic fermented goats’ milk decreases oxidative stress-mediated atherogenicity in human subjects. Br J Nutr.

[65] Zhang X-F, Guan X-X, Tang Y-J, Sun J-F, Wang X-K, Wang W-D (2021). Clinical effects and gut microbiota changes of using probiotics, prebiotics or synbiotics in inflammatory bowel disease: a systematic review and meta-analysis. Eur J Nutr.

[66] Abdollahi S, Meshkini F, Clark CC, Heshmati J, Soltani S (2022). The effect of probiotics/synbiotics supplementation on renal and liver biomarkers in patients with type 2 diabetes: a systematic review and meta-analysis of randomised controlled trials. Br J Nutr.

[67] Brantsæter AL, Myhre R, Haugen M, Myking S, Sengpiel V, Magnus P (2011). Intake of probiotic food and risk of preeclampsia in primiparous women: the Norwegian mother and child cohort study. Am J Epidemiol.

[68] North C, Venter C, Jerling J (2009). The effects of dietary fibre on C-reactive protein, an inflammation marker predicting cardiovascular disease. Eur J Clin Nutr.

[69] Lindsay KL, Kennelly M, Culliton M, Smith T, Maguire OC, Shanahan F (2014). Probiotics in obese pregnancy do not reduce maternal fasting glucose: a double-blind, placebo-controlled, randomized trial (probiotics in pregnancy study). Am J Clin Nutr.

[70] Collado MC, Isolauri E, Laitinen K, Salminen S (2008). Distinct composition of gut microbiota during pregnancy in overweight and normal-weight women. Am J Clin Nutr.

[71] Santacruz A, Collado MC, Garcia-Valdes L, Segura M, Martin-Lagos J, Anjos T (2010). Gut microbiota composition is associated with body weight, weight gain and biochemical parameters in pregnant women. Br J Nutr.

[72] Greiner T, Bäckhed F (2011). Effects of the gut microbiota on obesity and glucose homeostasis. Trends in Endocrinology & Metabolism.

[73] Zhang J, Ma S, Wu S, Guo C, Long S, Tan H. Effects of probiotic supplement in pregnant women with gestational diabetes mellitus: a systematic review and meta-analysis of randomized controlled trials. Journal of diabetes research. 2019;2019. 10.1155/2019/5364730 PMC6748202 31583250

[74] Motedayen M, Rafiei M, Tavirani MR, Sayehmiri K, Dousti M (2019). The relationship between body mass index and preeclampsia: a systematic review and meta-analysis. Int J Reproductive Biomed.

[75] Sfregola G, Sfregola P, Ruta F, Zendoli F, Musicco A, Garzon S (2023). Effect of maternal age and body mass index on induction of labor with oral misoprostol for premature rupture of membrane at term: a retrospective cross-sectional study. Open Med.

